# Cost-Effectiveness of Chuna Manual Therapy and Usual Care, Compared with Usual Care Only for People with Neck Pain following Traffic Accidents: A Multicenter Randomized Controlled Trial

**DOI:** 10.3390/ijerph18199994

**Published:** 2021-09-23

**Authors:** A-La Park, Eui-Hyoung Hwang, Man-Suk Hwang, In Heo, Sun-Young Park, Jun-Hwan Lee, In-Hyuk Ha, Jae-Heung Cho, Byung-Cheul Shin

**Affiliations:** 1 Care Policy and Evaluation Centre, Department of Health Policy, London School of Economics and Political Science, London WC2A 2AE, UK; 2 Department of Korean Medicine Rehabilitation, School of Korean Medicine, Pusan National University, Yangsan 50612, Korea; taichi20@gmail.com (E.-H.H.); hwangmansuk@pusan.ac.kr (M.-S.H.); higjdls@gmail.com (I.H.); shl0305@gmail.com (S.-Y.P.); drshinbc@gmail.com (B.-C.S.); 3 Spine and Joint Center, Pusan National University Korean Medicine Hospital, Yangsan 50612, Korea; 4 Clinical Medicine Division, Korea Institute of Oriental Medicine, Daejeon 34054, Korea; omdjun@kiom.re.kr; 5 Korean Medicine Life Science, Campus of Korea Institute of Oriental Medicine, University of Science & Technology (UST), Daejeon 34054, Korea; 6 Department of Korean Medicine Rehabilitation, Jaseng Hospital of Korean medicine, Seoul 02453, Korea; hanihata@gmail.com; 7 Department of Korean Medicine Rehabilitation, College of Korean Medicine, Kyung Hee University, Seoul 02447, Korea; vetkong95@hanmail.net

**Keywords:** cost-effectiveness, complementary and integrated medicine, Chuna manual therapy, neck pain, traffic accidents

## Abstract

This is the first cost-effectiveness analysis of Chuna manual therapy (CMT) plus usual Korean traditional medicine for traffic accident victims using a randomized controlled trial. A total of 132 participants were equally allocated to the intervention group receiving 6–11 sessions of CMT plus usual Korean traditional medicine care for three weeks or usual care including acupuncture, cupping, herbal medicine, moxibustion, and traditional physiotherapy at three hospitals. At 12 weeks, from a healthcare perspective, the intervention group had significantly higher costs (mean (SD), $778 (435) vs. $618 (318); difference, $160; 95% CI, $15 to $289; *p* = 0.005). From a societal perspective, total costs were insignificantly lower in the intervention group (mean (SD), $1077 (1081) vs. $1146 (1485); difference, $−69; 95% CI, $−568 to $377; *p* = 0.761). The intervention group dominated, with significantly higher QALYs gained at lower overall cost with a 72% chance of being cost-effective. From a societal perspective, the intervention was cost-saving for individuals who had neck pain after car accidents, although it was not cost-effective from the healthcare perspective ($40,038 per QALY gained). Findings support use of CMT as an integrated care treatment for whiplash from a societal perspective. Further studies with larger sample sizes are needed to determine cost-effectiveness in other cultural contexts.

## 1. Introduction

There can be substantial costs to individuals and families for healthcare, productivity losses, and informal care for Whiplash Associated Disorders (WAD). The annual costs of WAD in Europe are EUR 10 billion [1]. Insurance claim costs are considerable, as 60% of patients with acute WAD can develop chronicity [2], with lingering pain and deteriorating quality of life and employability [3]. One year after injury, 50% of people with WAD still experience neck pain due to subtle soft tissue damage that is not easily detected [4,5].

In 1999 in South Korea, the automobile compensation insurance scheme was expanded to include Korean traditional medicine, leading to an increase in use of complementary alternative medicine [6,7]. Approximately 30% of people with neck pain use manual therapies to manage their conditions [8] Patients without fractures and nerve damage tend to prefer Korean traditional medicine, as it is a less invasive, non-pharmacological approach. They are reluctant to use painkillers and painful injections [9,10]. A systematic review and meta-analysis of randomized clinical trials (RCTs) for Chuna (Tuina) manual therapy (CMT) showed significant reductions in musculoskeletal pain [11] and nonspecific chronic neck pain [12]. A recent RCT of CMT plus standard care for whiplash took significantly fewer days to reach a 50% reduction in pain, compared with standard care (23 vs. 50 days, *p* = 0.01) [13].

Despite increasingly positive clinical effectiveness studies, there are few economic evaluations of manual therapies for WAD/neck pain following car accidents worldwide. With increasing car insurance claims for WAD, it is important to look at cost-effectiveness of CMT as an adjunct therapy to usual care for WAD in a South Korean context to facilitate more optimal resource allocation decisions. This is the first cost-effectiveness analysis for CMT alongside a RCT in this population.

## 2. Materials and Methods

### 2.1. Trial Design and Participants

Patients aged 19–70 with whiplash lasting between two and 13 weeks following a traffic accident were recruited. Patients who consented, and did not have fractures or neurological damage, were allocated using block randomization (block size = 4) to CMT plus usual care or usual care only between August 2019 and February 2020 with 12-week follow-up ending in August 2020 (Figure S1). Neck pain was measured using a numeric rating scale (NRS) score ≥ 5 (moderate) and diagnosed with Whiplash Associated Disorders (WAD) grade I, II [14]. The study was approved by the ethics committees of the participating hospitals (Busan National University Korean Medicine Hospital-2019004, Kyung-Hee University Korean Medicine Hospital-KHNMCOH 2019-05-001-001, and Jaseng Hospital of Korean Medicine in Seoul-JASENG 2019-04-014-002). To estimate sample size, a previous clinical study that treated a pharmacopuncture (herbal acupuncture) treatment group, CMT only group, and combination pharmacopuncture and CMT treatment group two times per week for 4 weeks was used as a reference [15]. The minimum number of participants required for hypothesis testing was calculated using G*Power 3.1.9.2 for Windows (Heinrich-Heine-Universität, Düsseldorf, Germany) as 46 participants per group. Considering a 30% dropout rate, we enrolled 132 participants for the powered sample size.

### 2.2. Interventions

CMT can be defined as a Korean traditional medicine modality based on Tuina in Traditional Chinese Medicine along the meridians “integrating with modern scientific knowledge on anatomy, physiology, pathology from American chiropractic practice, osteopathy, and Japanese manipulation techniques, by correcting osteopathic structures using thrust, mobilization, distraction of the spine and joints, visceral manipulation and soft tissue release” [16]. Intervention consisted of 6 to 11 sessions of CMT plus usual care (UC). UC included acupuncture, cupping, moxibustion, herbal medicines, and physiotherapy. CMT was performed by Korean Medicine doctors with more than 3 years’ experience. The first session lasted 20 min, with subsequent treatments lasting 15 min.

### 2.3. Effectiveness

Impacts on quality of life were measured at baseline, 3, 6, and 12 weeks after intervention. Self-reported quality of life scores were measured using the EQ-5D-5L questionnaire [17,18]. Quality Adjusted Life years gained (QALYs) were calculated using the area under the curve method [19] at 12-week follow up.

### 2.4. Cost-Effectiveness Analysis

Cost-effectiveness analysis was conducted alongside the RCT and reported here as recommended in the Consolidated Health Economic Evaluation Reporting Standards (CHEERS) guidelines [20]. The evaluation was performed from both healthcare payer and societal perspectives. In South Korea, this evaluation will be of interest to healthcare providers such as hospitals, as well as car insurance providers, the major funders of healthcare services arising from traffic accidents. Data on prescribed medications, emergency visits, inpatient days in conventional western and traditional Korean Medicine hospitals, day care, outpatient care, and diagnostic tests such as X-rays were collected by patient self-report, using an adapted version of the Client Service Receipt Inventory (CSRI) [21] at baseline, 3, 6, and 12 weeks. This instrument is widely used to collect health services-related resource use through self-report. Intervention costs were estimated by examining electronic patient records.

Societal costs included out-of-pocket payments for over-the-counter food supplements (multivitamins, omega-3 fish oil, calcium, painkillers, collagen, and glucosamine). Travel-time costs for patients’ carers for Korean Medicine hospital visits were calculated using the official minimum wage rate of $10 per hour [22]. Transportation expenses were estimated for use of private and public transport. Absenteeism and informal care costs were estimated using a human capital approach, assuming each day absent from work or providing informal care was equivalent to 8 h on minimum wage.

Relevant unit costs from the national automobile insurance providers association were applied to all health resources used (Tables S3–S5) [23]. All costs were converted from South Korean Won (2019) to 2020 purchasing power parity (PPP) international dollars ($), using the IMF currency conversion and inflation rates [24].

### 2.5. Statistical Analysis

Economic analyses were performed using the intention-to-treat principle. Cost comparisons were made at four timepoints over 12 weeks. Baseline costs included costs for health services and societal costs. Independent *t*-tests were performed and 95% confidence intervals estimated using bias-corrected accelerated bootstrapping, given skewed cost distributions. 

Incremental cost per QALY gained, using the net-benefit approach [25], was estimated. Discounting was not performed as the intervention lasted less than one year. In sensitivity analyses, alternative scenarios explored whether results remained robust by varying intervention costs and effects, and including imputed values.

In cost-effectiveness planes, joint distributions of incremental mean costs and QALYs for CMT+UC versus UC groups were generated using non-parametric bootstrapping with 1000 replications to explore the probability of intervention being a better option at the willingness-to-pay (WTP) level set by funders for improved quality of life in South Korea. Cost-effectiveness acceptability curves were generated by plotting these probabilities at various WTP values. Missing costs and outcomes were imputed using multiple imputation with predictive mean matching to deal with skewness [26,27]. All analyses were 2-tailed, and a *p*-value of < 0.05 was considered significant. Data were analyzed using SPSS 24 [28] and STATA 15 [29].

## 3. Results

### 3.1. Effectiveness

A total of 132 participants (mean (SD) age, 40.0 (11.8) years; 91 (69%) women and 41 (31%) men) were allocated evenly to CMT+UC or UC groups. There were no significant differences in age, gender, height, weight, and BMI at baseline. (See baseline characteristics in Table S1). At a 12-week follow-up the CMT+UC group showed significantly better quality of life than the UC group (mean (SD) QALY gained per person, 0.193 (0.010) vs. 0.189 (0.010); mean difference, 0.004; 95% CI, 0.0002 to 0.007; *p* *=* 0.037) (see Table S2). 

### 3.2. Intervention Costs

The CMT+UC group had significantly higher intervention costs, compared with the UC only group (mean (SD), $639 (214) vs. $462 (136); difference, $177; 95% CI, $112 to $236; *p* = 0.001). No significant difference was found for UC costs between the two groups (mean (SD), $457 (160) vs. $458 (130); difference, $−1; 95% CI, $−49 to $58; *p* = 0.097). This indicates randomization between the two groups was successful, implying the incremental cost difference can be attributed to additional Chuna costs.

### 3.3. Health Service Costs

In Table 1, baseline costs were used to check whether there were statistically significant differences in costs before receipt of treatment; no differences were found. Thus, any cost differences incurred after intervention can be attributed to the difference made as a result of intervention in the RCT. Baseline inpatient costs at Korean Medicine hospitals for CMT+UC group were non-significantly higher than the UC group by $25 (mean (SD), $463 (970) vs. $438 (815); mean difference, $24; 95% CI, −285 to 338; *p* = 0.88). 

Emergency care costs were lower in the CMT+UC group. No inpatient care costs in conventional western or traditional Korean medicine hospitals were incurred during follow-up. This may be as patients had relatively minor to moderate neck injuries (Whiplash Associated Disorder grade 1 or 2). There were no daycare costs. Costs for outpatient care and diagnostic tests were less costly at 3-weeks and 12-weeks in the CMT+UC versus UC group, while slightly higher at 6 weeks. 

Overall, healthcare costs, excluding intervention costs, were lower for the CMT+UC group (Mean (SD), $148 (340) vs. $164 (292); mean difference, $−16; 95% CI, −115 to 84; *p* = 0.785). When including intervention costs, healthcare costs were significantly higher by $160 (Mean (SD), $778 (435) vs. $618 (318); mean difference, $160; 95% CI, 15 to 289; *p* = 0.005).

### 3.4. Societal Costs

Societal costs at four different timepoints are reported in Table 2. From this perspective, there were no significant differences in costs at baseline. Productivity losses due to sickness absence by patients were consistently higher in the UC group from baseline until 12-weeks, although this was not significant. No informal care costs were incurred, as patients had relatively minor neck injuries, but this could be due to cultural perception, as patients may not have considered help received, such as support with daily activities of living (e.g., cooking, shopping, and cleaning) from family or friends as “extra” support.

Overall, societal costs over 12 weeks, excluding intervention costs, were non-significantly lower in the CMT+UC group (Mean (SD), $466 (994) vs. $716 (1486); mean difference, $−250; 95% CI, −715 to 178; *p* = 0.317). Similarly, after including intervention costs, accumulated societal costs were still non-significantly lower by $69 (Mean (SD), 1077 (1081) vs. 1146 (1485); mean difference, $−69; 95% CI, −568 to 377; *p* = 0.761).

### 3.5. Incremental Cost-Effectiveness Ratio

From the healthcare perspective, the ICER, calculated as incremental costs divided by incremental effects is $40,038 per QALY. This is higher than the accepted WTP level of 20,000,000 Won ($24,656) per QALY in South Korea. From a societal perspective, with no significant difference in costs and better outcomes, the CMT+UC group is considered the dominant option and, by convention, the ICER is not reported. 

### 3.6. Sensitivity Analyses

Sensitivity analyses were performed to explore impacts of varying assumptions. We examined using a minimum of just one Chuna session over the 3-week intervention period, instead of at least 2 sessions per week over 3-weeks. Interestingly, although costs in the CMT+UC group were $11 higher than the UC group (Mean (SD), 168 (332) vs. 157 (287); mean difference, $11; 95% CI, −94 to 124; *p* = 0.86), the cost difference was no longer significant from the healthcare perspective. From a societal perspective, costs decreased by $217 (Mean (SD), 467 (970) vs. 684 (1459); mean difference, $−217; 95% CI, −707 to 219; *p* = 0.32).

Using imputed costs did not change the final conclusions, given the small percentage (8–9%) of missing data in both arms. Imputed total healthcare costs were significantly more expensive in the CMT+UC versus UC group, due to the extra cost of CMT (Mean (SD), 796 (416) vs. 633 (307); mean difference, $163; 95% CI, 30 to 288; *p* = 0.01). Imputed total societal costs were less in the CMT+UC versus UC group (Mean (SD), 1137 (1051) vs. 1193 (1456); mean difference, $−56; 95% CI, −550 to 411; *p* = 0.80). Sensitivity analyses showed no major impact on interpretation of findings.

### 3.7. Cost-Effectiveness Planes

**Figure 1:**
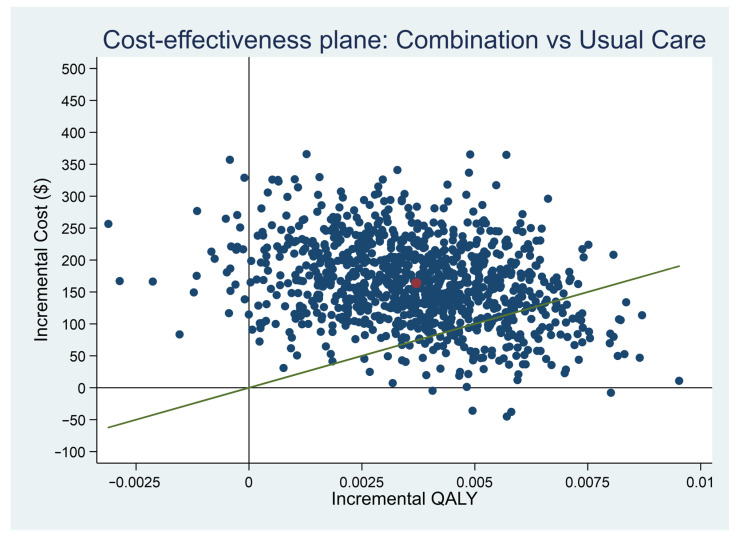
Cost–Effectiveness Plane (healthcare payer perspective). Red dot: Incremental cost-effectiveness ratio, Green line: willingness-to-pay threshold.

Figure 1 shows the cost-effectiveness plane from the healthcare payer perspective. Most simulated paired dots, after bootstrapping 1000 times, were scattered above the accepted threshold line of 20,000,000 won ($24,656) (green line) in the north-east quadrant. This means CMT+UC was more costly than UC, but more effective. In this case, cost-effectiveness can be determined using the threshold value. The ICER (red dot) is above the threshold, indicating that CMT+UC was not cost-effective from the healthcare perspective. 

**Figure 2:**
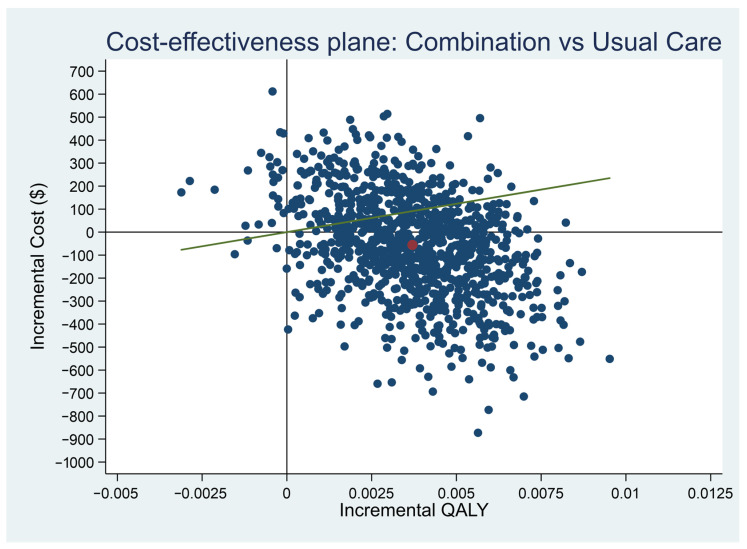
Cost–Effectiveness Plane (societal perspective). Red dot: Incremental cost-effectiveness ratio, Green line: willingness-to-pay threshold.

In contrast, from the societal perspective, the majority of paired dots after bootstrapping 1000 times, were scattered below the threshold value. The ICER (red dot) was also located in the south-east quadrant in Figure 2. This indicated CMT+UC was less costly and more effective (i.e., better quality of life) in the simulated cost-effectiveness plane, compared with usual care only.

### 3.8. Cost-Effectiveness Acceptability Curves

**Figure 3:**
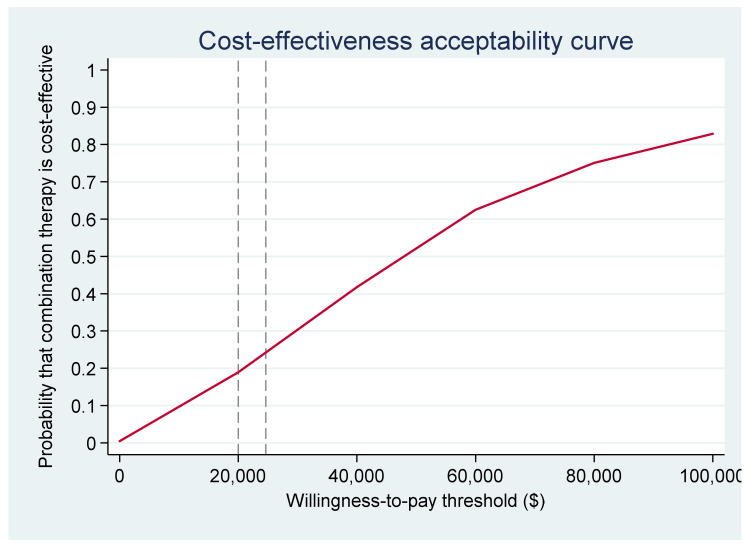
Cost–Effectiveness Acceptability Curve (healthcare payer perspective).

**Figure 4:**
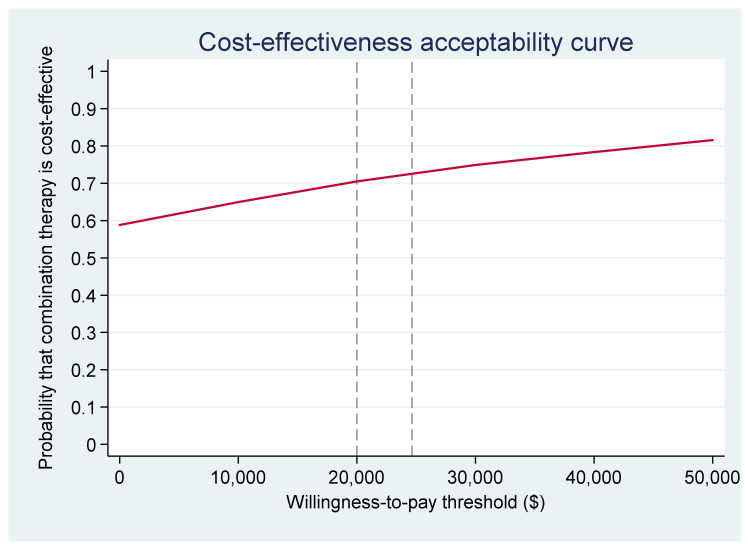
Cost–Effectiveness Acceptability Curve (societal perspective).

In Figure 3, the cost-effectiveness acceptability curve from the health payer perspective, indicates only a 24% chance of CMT+UC being cost-effective at the threshold value. However, in Figure 4 from the societal perspective there was a 72% chance of CMT+UC being cost-effective, with costs in the CMT+UC group likely to be lower than costs in the UC group. 

## 4. Discussion

From a societal perspective, the probability that CMT+UC was cost saving at 12-weeks was very high, even at very low levels of willingness to pay. Most cost savings were driven by reductions in productivity loss from sickness absence and travel-time transportation costs in the CMT+UC group. Depending on the perspective of the economic analysis, the results differ, as it was unlikely to be cost-effective from a healthcare payer perspective. Therefore, it is important to consider a broad societal perspective that captures more of the full costs and benefits to society, otherwise we might dismiss promising interventions. After all, payers to car insurance companies and taxpayers to the healthcare system are individual service users.

The lack of significant difference in most health service and societal costs could be attributed to the lack of power to detect significant differences at four time points due to the small sample size. It is also worth noting that greater sample sizes are generally needed to detect a significant difference in costs compared to effects, although in practice, power calculations for RCTs are usually based on primary clinical outcomes rather than costs [19]. Therefore, further RCTs with larger sample sizes might be helpful to confirm differences in costs are significant.

### 4.1. Generalizability of Findings

This study was conducted in South Korea, a country where CMT is commonly provided within the integrated healthcare system and used by the general population seeking help for pain management. Chuna has been included within the traditional Korean Medicine standard care package reimbursed by automobile insurance providers since 1999. 

South Korea has a well-integrated health system combining CAM modalities and western medicine. Various traditional Korean medicine modalities for pain management are provided as a part of the standard care package for victims of road traffic accidents including whiplash injuries. Moreover, health care costs after car accidents are covered entirely by car insurance companies, and insurance is mandatory for every driver under the Korean Automobile Act [30]. Private car insurance companies and medical institutions directly pay these treatment costs, and patients are not usually directly involved in the transaction process. Although no direct payments for treatment by patients may appear to be a moral hazard, encouraging patients to overconsume services, there is a cap on care set by car insurance companies. Patients can choose their preferred services within this cap, depending on clinical needs, in consultation with medical professionals. In addition, South Korea has a high number of Korean Medicine practitioners in private practice compared with CAM practitioners in other western countries. Although provision of CMT to patients with neck pain after automobile accidents may not be directly comparable, additional provision of manual therapy as part of multimodal care can be comparable to practice in other western countries [31]. 

In previous studies, cost-effectiveness was more favorable to manual therapies when economic evaluations were conducted from societal rather than healthcare payer perspectives. In the UK, from the National Health Service (NHS) payer perspective, physiotherapist manual therapy, in addition to advice and exercise, was not cost-effective, compared with advice and personalized exercise, being more costly ($239 vs. $211) and less effective (0.342 vs. 0.362) [32]. However, from a societal perspective, manual therapy became cost-effective at $19,279 per QALY, an acceptable threshold value.

Similarly, in another UK NHS study, usual physiotherapy, including manual therapy, acupuncture, electrotherapy, and advice, relative to brief intervention by physiotherapists via self-care and cognitive behavioral therapies was not cost-effective at $142,892 per QALY gained [33] Other studies conducted from a societal perspective showed more favorable results. Manual therapy including manipulation, mobilization, advice, and exercise was dominant, compared to behavioral graded activity, aiming to increase the activity levels over time by physiotherapists [34]. In a Dutch study, manual therapy for muscular mobilization and stabilization was found to be a dominant strategy, compared with usual physiotherapy including massage, relaxation techniques, and traction. Manual therapy was also dominant, relative to usual care by general practitioners including advice on good posture, educational leaflets on self-care and anti-inflammatory medications [35].

Therefore, our cost-effectiveness study results are consistent with general trends in existing studies, in that the broader the economic perspective, the more cost-effective manual therapy is when provided in combination with various therapies and/or usual care. Reduced costs associated with productivity losses and better quality of life from manual therapy can potentially generate cost-savings to society as a whole. More studies are needed to confirm generalizability internationally in different socio-cultural contexts.

### 4.2. Strengths

To our best knowledge, our present study is the first cost-effectiveness analyses alongside an RCT for CMT in patients with whiplash after traffic accidents, although we have highlighted economic evaluations for non-invasive manual therapies for neck pain associated disorders. Given the lack of economic evidence on manual therapies for people experiencing neck pain after car accidents worldwide, our study will contribute to bridging the gap in research in this field. Economic evidence can facilitate relevant stakeholders, such as funders and healthcare professionals, to make optimal decisions on scarce resource allocation, as CMT is non-invasive and improves quality of life. From the healthcare payer/car insurance perspective, it was more expensive than UC, but is a dominant strategy, potentially cost-saving at a societal level. Results remained robust in sensitivity analyses. 

### 4.3. Limitations

We did not have access to electronic records on the wider use of health services by patients or on their productivity losses and instead relied on patient recall. However, to minimize any risk of bias, we adapted a validated and widely used approach to collecting self-report data, the CSRI [21]. In health economic evaluations, it is very well established and well accepted to ask patients for the service use for the past 3 months or less than 3 months, without recall bias being a substantial concern [36]. In our study, as the time periods (3 weeks, 6 weeks) were much shorter than the typically accepted 3 month recall period, we do not believe recall bias is a significant concern. 

In our analysis there did not appear to be any informal care costs associated with injury, however this might be an underestimation of the costs associated with the actual informal care provided by carers, as respondents might have considered caring as a culturally moral duty rather than as an “extra” caring activity. We might have addressed this issue by providing some illustrative examples of what we meant by informal care, such as helping with daily activities of living (e.g., preparing extra meals, grocery shopping, monitoring medication times, paying bills, etc.). This might have helped respondents to have better understanding of caring activities in the resource use questionnaire. 

We also conservatively did not ask about productivity losses that were not related to work. However, not everyone who is injured in a car-accident will be in paid employment, but they can still incur opportunity costs related to unpaid productive activities. For example, their own caring responsibilities (e.g., looking after children or parents) may be affected. There are also potential opportunity costs associated with lost unpaid productive activities, such as volunteering in the local community. These might be considered in future studies.

Considering implementation in other countries, the intervention may not be feasible due to differences in treatment costs covered by healthcare systems and availability of trained therapists. Potentially, it could be considered for medical training curriculums and/or training programs for physiotherapists, depending on whether there is demand from patients as an add-on therapy in outpatient settings as part of care pathways, given improved productivity and quality of life outcomes. Future economic studies can also explore the economic consequences of using well-being measures, given the non-invasive therapeutic nature of CMT. The potential for cost-saving would be even greater when tested in RCTs with larger sample sizes and longer follow-up periods.

## 5. Conclusions

From a societal perspective, adding CMT to usual care is potentially cost-saving, while significantly improving quality of life in patients with whiplash following car accidents. The results suggest Chuna can be an optional add-on therapy. More economic studies with larger sample sizes might be useful to help determine cost-effectiveness of CMT internationally, taking into account the extent to which integrated care modalities are incorporated into usual care in various health systems and cultural settings.

## Figures and Tables

**Table 1:** Health care costs at four timepoints.

		Chuna+Usual Care			Usual Care			Difference	BCa 95% Confidence Interval		
Cost Category	Time	N	Mean	SD	N	Mean	SD	Mean	Lower	Upper	*p*-Value
Drug cost	Baseline	66	5	11	65	7	13	−2	−6	2	0.31
	3 weeks	61	0	2	61	0	1	0	0	1	0.999
	6 weeks	61	1	2	60	0	1	0	0	1	0.41
	12 weeks	58	1	4	62	1	3	0	−1	2	0.53
Emergency	Baseline	66	43	61	65	52	72	−9	−30	13	0.46
	3 weeks	61	2	17	61	2	17	0	−6	6	0.999
	6 weeks	61	0	0	60	0	0	0	.	.	.
	12 weeks	58	0	0	62	2	16	−2	−8	2	0.12
Inpatient care at conventional hospitals	Baseline	66	176	503	66	298	608	−122	−312	85	0.21
	3 weeks	61	0	0	61	0	0	0	.	.	.
	6 weeks	61	0	0	60	0	0	0	.	.	.
	12 weeks	58	0	0	62	0	0	0	.	.	.
Inpatient care at Korean Medicine hospitals	Baseline	66	463	970	66	438	815	24	−285	338	0.88
	3 weeks	61	0	0	61	0	0	0	.	.	.
	6 weeks	61	0	0	60	0	0	0	.	.	.
	12 weeks	58	0	0	62	0	0	0	.	.	.
Outpatient	Baseline	66	317	385	66	335	438	−18	−149	127	0.80
	3 weeks	61	47	121	61	48	120	−1	−46	39	0.96
	6 weeks	61	46	105	60	40	93	6	−30	43	0.75
	12 weeks	58	53	245	62	72	183	−19	−89	72	0.64
Diagnostic tests	Baseline	66	15	21	66	17	22	−3	−11	5	0.46
	3 weeks	61	1	3	61	1	4	0	−2	1	0.76
	6 weeks	61	1	5	60	0	2	1	0	2	0.32
	12 weeks	58	1	5	62	1	7	0	−3	2	0.75

Abbreviations: SD, standard deviation; Bca: biased-corrected accelerated bootstrap.

**Table 2:** Societal costs at four time-points.

		Chuna+Usual Care			Usual Care			Difference	BCa 95% Confidence Interval		
Cost Category	Time	N	Mean	SD	N	Mean	SD	Mean	Lower	Upper	*p*-Value
Food supplements	Baseline	66	5	20	66	9	32	−4	−14	5	0.44
	3 weeks	61	3	11	61	5	21	−2	−9	3	0.47
	6 weeks	61	1	6	60	5	27	−4	−12	1	0.36
	12 weeks	58	5	20	60	2	8	3	−1	9	0.27
Travelling costs	Baseline	54	19	8	44	21	7	−2	−5	1	0.14
	3 weeks	61	32	16	61	34	22	−2	−9	5	0.56
	6 weeks	61	12	29	60	8	25	4	−6	14	0.41
	12 weeks	58	12	49	60	13	32	−1	−16	17	0.87
Transportation costs	Baseline	66	72	348	66	43	81	29	−27	130	0.57
	3 weeks	61	4	13	61	8	35	−4	−16	5	0.46
	6 weeks	61	6	23	60	5	14	1	−5	8	0.88
	12 weeks	58	3	10	60	13	36	−10	−20	−2	0.07
Absenteeism costs	Baseline	45	425	612	42	710	852	−285	−608	18	0.09
	3 weeks	61	31	172	61	94	342	−63	−169	31	0.22
	6 weeks	61	43	253	60	82	296	−38	−141	67	0.45
	12 weeks	58	30	227	60	142	633	−112	−313	54	0.26

SD: standard deviation, BCa: bias-corrected and accelerated bootstrap.

## Data Availability

Not Applicable.

## Supplementary Materials

The following are available online at https://www.mdpi.com/article/10.3390/ijerph18199994/s1, Figure S1: S1. CONSORT flow diagram, Table S1: Baseline characteristics of participants, Table S2: EQ-5D-5L utility values at 4 timepoints, Table S3: Unit costs, Table S4: Health care service utilization at 4 timepoints, Table S5: Number of hours lost due to travelling by carers and absenteeism by patients.
