# Supplementary material for: Cost-Effectiveness of Chuna Manual Therapy and Usual Care, Compared with Usual Care Only for People with Neck Pain following Traffic Accidents: A Multicenter Randomized Controlled Trial
Source: Int J Environ Res Public Health. 2021 Sep 23;18(19):9994. doi: 10.3390/ijerph18199994 (PMC8508460; doi:10.3390/ijerph18199994)
Supplement: Supplementary file 1 [file ijerph-18-09994-s001.zip › ijerph-1348440-supplementary.pdf]

Figure S1. CONSORT flow diagram

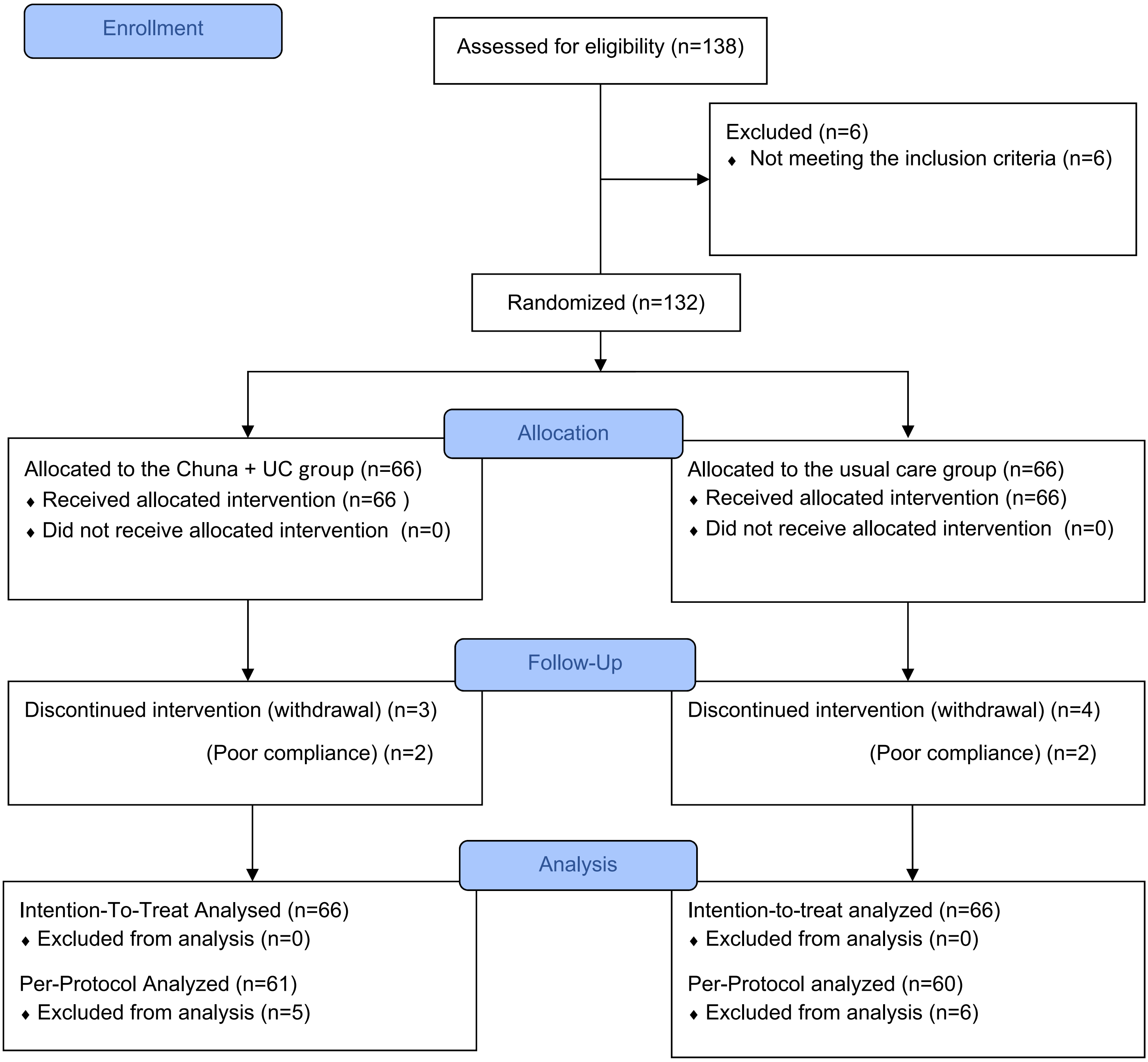

Supplementary Tables (Table S1, S2, S3, S4, S5)

Table S1. Baseline characteristics of participants.

Variables		UC alone (n = 66)	CMT+UC (n = 66)	p value
SEX N (%)	Male	24 (36.4)	17 (25.8)	.188[1)]
	Female	42 (63.6)	49 (74.2)	
Age (years)		40.30 ± 12.13	39.61 ± 11.55	.736[2)]
Height (cm)		166.02 ± 8.39	165.20 ± 7.80	.563[2)]
Weight (kg)		66.12 ± 12.93	62.63 ± 12.11	.112[2)]
BMI		24.12 ± 3.73	22.88 ± 3.56	.053[2)]

[1)] P values were derived from the chi-square test.
[2)] P values were derived from an independent t test for between-group comparisons.
BMI, body mass index; CMT, Chuna manual treatment; UC, usual care

Table S2. EQ-5D-5L utility values at 4 timepoints.

EQ-5D	Chuna+Usual Care			Usual Care			Difference	BCa 95% CI		
Time	N	Mean	SD	N	Mean	SD	Mean	Lower	Upper	P-value
Baseline	61	0.799	0.049	60	0.801	0.048	-0.002	-0.019	0.013	0.787
3 weeks	61	0.828	0.044	60	0.812	0.05	0.017	0.000	0.031	0.050
6 weeks	61	0.835	0.053	60	0.821	0.048	0.014	-0.003	0.03	0.103
12 weeks	61	0.858	0.046	60	0.828	0.052	0.03	0.013	0.045	0.001

SD: standard deviation; BCa 95% CI: bias-corrected accelerated bootstrap 95% Confidence Interval

Table S3: Unit costs

Cost category	Unit costs (Int$)	Source
Drug cost per day	5.39	[23]
Emergency per day	129	[23]
Inpatient care at conventional hospitals per day	177.97	[23]
Inpatient care at Korean Medicine hospitals per day	177.97	[23]
Outpatient per day	65.45	[23]
Diagnostic tests per day	12.29	[23]
Minimum wage per hour	10	[22]

Table S4: Health care service utilization at 4 timepoints

		Chuna+Usual Care			Usual Care			Difference	BCa 95% CI	
Cost category	Time	N	Mean	SD	N	Mean	SD	Mean	Lower	Upper
Drug	Baseline	66	0.83	2.00	65	1.23	2.41	-0.40	-1.11	0.29
	3 weeks	61	0.07	0.31	61	0.07	0.25	0.00	-0.09	0.10
	6 weeks	61	0.10	0.40	60	0.05	0.22	0.05	-0.06	0.16
	12 weeks	58	0.19	0.74	62	0.11	0.58	0.08	-0.16	0.34

Emergency	Baseline	66	0.33	0.48	65	0.40	0.55	-0.07	-0.24	0.10
	3 weeks	61	0.02	0.13	61	0.02	0.13	0.00	-0.05	0.05
	6 weeks	61	0.00	0.00	60	0.00	0.00	0.00	.	.
	12 weeks	58	0.00	0.00	62	0.02	0.13	-0.02	-0.06	0.01
Inpatient care at conventional hospitals	Baseline	66	0.98	2.81	66	2.30	3.41	-0.68	-1.75	0.48
	3 weeks	61	0.00	0.00	61	0.00	0.00	0.00	.	.
	6 weeks	61	0.00	0.00	60	0.00	0.00	0.00	.	.
	12 weeks	58	0.00	0.00	62	0.00	0.00	0.00	.	.
Inpatient care at Korean Medicine hospitals	Baseline	66	2.59	5.43	66	2.45	4.56	0.14	-1.60	1.89
	3 weeks	61	0.00	0.00	61	0.00	0.00	0.00	.	.
	6 weeks	61	0.00	0.00	60	0.00	0.00	0.00	.	.
	12 weeks	58	0.00	0.00	62	0.00	0.00	0.00	.	.
Outpatient	Baseline	66	4.83	5.87	66	5.11	6.67	-0.27	-2.27	1.93
	3 weeks	61	0.72	1.85	61	0.74	1.83	-0.02	-0.70	0.60
	6 weeks	61	0.70	1.60	60	0.62	1.42	0.09	-0.46	0.65
	12 weeks	58	0.81	3.73	62	1.10	2.78	-0.29	-1.36	1.10
Diagnostic test	Baseline	66	1.18	1.71	66	1.41	1.79	-0.23	-0.86	0.39
	3 weeks	61	0.07	0.25	61	0.08	0.33	-0.02	-0.12	0.08
	6 weeks	61	0.08	0.42	60	0.02	0.13	0.07	-0.03	0.18
	12 weeks	58	0.07	0.41	62	0.10	0.53	-0.03	-0.22	0.13

Table S5: Number of hours lost due to travelling by carers and absenteeism by patients

		**Chuna+Usual Care**			**Usual Care**			**Difference**	**BCa 95% CI**	
**Cost category**	**Time**	**N**	**Mean**	**SD**	**N**	**Mean**	**SD**	**Mean**	**Lower**	**Upper**
Travelling time	Baseline	54	1.85	0.76	44	2.07	0.70	-0.22	-0.51	0.07
	3 weeks	61	3.10	1.59	61	3.30	2.16	-0.19	-0.86	0.46
	6 weeks	61	1.17	2.79	60	0.77	2.40	0.41	-0.57	1.35
	12 weeks	58	1.15	4.80	60	1.29	3.09	-0.14	-1.53	1.69
Absenteeism	Baseline	45	41.24	59.48	42	68.95	82.78	-27.71	-59.06	1.75
	3 weeks	61	3.02	16.66	61	9.11	33.23	-6.10	-16.41	2.99
	6 weeks	61	4.20	24.60	60	7.93	28.75	-3.74	-13.66	6.46
	12 weeks	58	2.90	22.06	60	13.81	61.53	-10.91	-30.38	5.23
